# Blood *FOLR3* methylation dysregulations and heterogeneity in non-small lung cancer highlight its strong associations with lung squamous carcinoma

**DOI:** 10.1186/s12931-024-02691-8

**Published:** 2024-01-25

**Authors:** Yunhui Qu, Xiuzhi Zhang, Rong Qiao, Feifei Di, Yakang Song, Jun Wang, Longtao Ji, Jie Zhang, Wanjian Gu, Yifei Fang, Baohui Han, Rongxi Yang, Liping Dai, Songyun Ouyang

**Affiliations:** 1 Department of Clinical Laboratory, the First Affiliated Hospital of Zhengzhou University and the Key Clinical Laboratory of Henan Province, Zhengzhou, 450052 China; 2 Department of Epidemiology, School of Public Health, Zhengzhou University, Zhengzhou, 4500001 China; 3 Department of Pulmonary Medicine, Shanghai Chest Hospital, Shanghai Jiaotong University, Shanghai, 200030 China; 4 Nanjing TANTICA Biotechnology Co. Ltd, Nanjing, 210000 China; 5 Henan Institute of Medical and Pharmaceutical Sciences & Henan Key Medical Laboratory of Tumor Molecular Biomarkers, Zhengzhou University, Zhengzhou, 450052 China; 6 Department of Clinical Laboratory, Jiangsu Province Hospital of Chinese Medicine, Nanjing, 210000 China; 7 Department of Respiratory and Sleep Medicine, the First Affiliated Hospital of Zhengzhou University, Zhengzhou, 450052 China; 8 Department of Epidemiology, School of Public Health, Nanjing Medical University, Nanjing, 210000 China

**Keywords:** Lung cancer, Early detection, DNA methylation, *FOLR3*, Mass spectrometry

## Abstract

**Background:**

Non-small cell lung cancer (NSCLC) accounts for the vast majority of lung cancers. Early detection is crucial to reduce lung cancer-related mortality. Aberrant DNA methylation occurs early during carcinogenesis and can be detected in blood. It is essential to investigate the dysregulated blood methylation markers for early diagnosis of NSCLC.

**Methods:**

NSCLC-associated methylation gene folate receptor gamma (*FOLR3*) was selected from an Illumina 850K array analysis of peripheral blood samples. Mass spectrometry was used for validation in two independent case–control studies (validation I: n = 2548; validation II: n = 3866). Patients with lung squamous carcinoma (LUSC) or lung adenocarcinoma (LUAD), normal controls (NCs) and benign pulmonary nodule (BPN) cases were included. *FOLR3* methylations were compared among different populations. Their associations with NSCLC clinical features were investigated. Receiver operating characteristic analyses, Kruskal–Wallis test, Wilcoxon test, logistics regression analysis and nomogram analysis were performed.

**Results:**

Two CpG sites (CpG_1 and CpG_2) of *FOLR3* was significantly lower methylated in NSCLC patients than NCs in the discovery round. In the two validations, both LUSC and LUAD patients presented significant *FOLR3* hypomethylations. LUSC patients were highlighted to have significantly lower methylation levels of CpG_1 and CpG_2 than BPN cases and LUAD patients. Both in the two validations, CpG_1 methylation and CpG_2 methylation could discriminate LUSC from NCs well, with areas under the curve (AUCs) of 0.818 and 0.832 in validation I, and 0.789 and 0.780 in validation II. They could also differentiate LUAD from NCs, but with lower efficiency. CpG_1 and CpG_2 methylations could also discriminate LUSC from BPNs well individually in the two validations. With the combined dataset of two validations, the independent associations of age, gender, and *FOLR3* methylation with LUSC and LUAD risk were shown and the age-gender-CpG_1 signature could discriminate LUSC and LUAD from NCs and BPNs, with higher efficiency for LUSC.

**Conclusions:**

Blood-based *FOLR3* hypomethylation was shown in LUSC and LUAD. *FOLR3* methylation heterogeneity between LUSC and LUAD highlighted its stronger associations with LUSC. *FOLR3* methylation and the age-gender-CpG_1 signature might be novel diagnostic markers for the early detection of NSCLC, especially for LUSC.

**Supplementary Information:**

The online version contains supplementary material available at 10.1186/s12931-024-02691-8.

## Introduction

Lung cancer (LC) is a malignant tumor occurring in the glands or bronchial mucosas. Pathologically, LC is mainly classified into two major subtypes, small cell lung cancer (SCLC) and non-small cell lung cancer (NSCLC). NSCLC accounts for 80–85% of all LC cases, of which the most common types are lung adenocarcinoma (LUAD) and lung squamous cell carcinoma (LUSC) [1]. As the leading cause of cancer-related mortality in the world [2], the prognosis of LC is highly correlated with the stage at initial diagnosis. The 5-year survival rate of LC patients at stage I is 83%, while decreases to 6% for stage IV patients [3]. The poor prognosis of LC patients is mainly due to the initial diagnosis at advanced-stage [4]. Thus, early detection is important for better treatment of the patients.

The screening of persons at high risk for LC by low-dose computed tomographic (LDCT) has presented an inspiring 20.0% decrease in mortality of LC in a large randomized controlled trial [5]. Although LDCT has shown a sensitivity of 93.7% for LC screening in high-risk populations (55–75 years old, > 30 packs of cigarettes per year) [5], it has a dramatically high false positive rate of 96.4% for distinguishing the malignant nodules from benign nodules [6]. When LDCT is applied for the screening program of general populations, the specificity will be even lower. A lot of effort has been made to search for molecular biomarkers in LC. For instance, the somatic mutations in *CXCR2* [7], *EGFR* [8, 9] and *DDR2 *[8] are involved in the pathogenesis of LC. Methylations of several genes including *ALDH2* [10], APC [11], *CDO1*, and *GSHR* [12] have been also reported to be associated with LC. The serum concentrations of CEA, CA125 and CYFRA are identified as prognostic markers in NSCLC [13, 14]. However, due to low sensitivity and/or specificity, these molecular methods can hardly be applied for the early detection of the patients.

Aberrant epigenetic change is a ubiquitous feature of carcinogenesis and often occurs in the early stage [15]. Hypermethylation of tumor suppressor genes and hypomethylation of oncogenes are early events in many cancers, suggesting altered DNA methylation patterns as one of the first detectable changes during tumorigenesis [15, 16]. Altered cfDNA methylation in the plasma has been identified in multiple cancers [17], but its limitations for early detection can’t be ignored, including low quantity of tumor DNA in the plasma at early stage, low sensitivity and high costs with very deep sequencing [18, 19]. Recent studies have suggested that the DNA methylation signatures in the peripheral blood could be efficient biomarkers for the early detection of cancer even at preclinical stage [20–22]. However, most of these studies were preliminary and mostly from a single clinical center with limited sample size.

In this study, Illumina 850K methylation array was conducted to screen for NSCLC-related DNA methylation alterations in peripheral blood. The selected candidate methylation gene *FOLR3* were further validated in two independent case–control studies by mass spectrometry. The correlations between *FOLR3* methylation and the clinical characteristics of LUSC and LUAD were also investigated. The diagnostic power of *FOLR3* methylations were evaluated. With age, gender, and *FOLR3* methylation, LUSC and LUAD risk models were constructed and their diagnostic potential were shown. We hope these results would provide new directions for early detection of LC, especially for NSCLC.

## Materials and methods

### Study populations

This study was approved by the Ethics Committee of all clinical centers following the Declaration of Helsinki (approve ID: KS1407 in Shanghai Chest Hospital and approve ID: 2021-KY-1057-002 in the First Affiliated Hospital of Zhengzhou University; The Jiangsu Province Hospital of Chinese Medicine is an organization of exemption from ethical approval). The written informed consents have been collected from all the recruited participants. The diagnosis of LC was confirmed by thoracic surgery and tissue pathology, and the blood samples were collected before surgery and any cancer-related treatments. A total of 741 NSCLC patients and 204 cases with benign pulmonary nodules (BPN) were recruited from Shanghai Chest Hospital (validation I) from 2020 to 2021. The 1230 NSCLC patients and 299 patients with BPNs in validation II were collected at the First Affiliated Hospital of Zhengzhou University. All the normal controls (NCs) (validation I: n = 1603, validation II: n = 2361) were obtained from the Jiangsu Province Hospital of Chinese Medicine. The inclusion criteria for the NSCLC patients and BPN cases was: (1) adult patients ≥ 18 years old and able to provide written informed consent; (2) single or multiple pulmonary nodules detected by LDCT screening; (3) a high suspicion of LC or BPN by clinical and/or imaging assessment, with planned biopsy or surgical resection for confirming diagnosis within two month after drawing blood; (4) blood samples could be collected prior to any treatment including local/regional therapy, radiation, systemic chemotherapy or surgery. Exclusion criteria: (1) pregnant or lactating; (2) participants who were ever diagnosed with any other cancer; (3) participants who had received organ transplantation or allogeneic hematopoietic stem cell transplantation. All the enrolled patients of LC or BPN cases underwent thoracic surgery and pathological examination. Pathological stages of all LC cases were determined by the doctors based on the 8th edition of the American Joint Committee on Cancer (AJCC) classifications. The inclusion criteria for NCs were: age ≥ 18 years old; with no cancer and cancer history; with no inflammatory disease; with no pulmonary nodules. Only the subjects conformed to all the items of the inclusion criteria were included, otherwise, they would be excluded. The clinical characteristics of samples were shown in Additional file 1: Table S1. All the NCs had normal blood accounts. None of the BPN cases and NCs had LC history. The processes of drawing and storing the blood samples in two validations were consistent.

### Sample processing

All the peripheral blood samples were collected by ethylene diamine tetraacetic acid (EDTA) blood collection tubes, and stored at – 80 ℃ till usage. All samples were randomized and processed double-blinded. DNA was extracted from blood by the DNA Extraction Kit (TANTICA, Nanjing, China), and further bisulfite-converted by DNA Methylation Gold Kit (TANTICA, Nanjing, China).

### Illumina 850K methylation assay

In the discovery study, bisulfite converted DNA from each sample was subjected to the genome-wide DNA methylation profiling using the Illumina Infinium Human Methylation EPIC 850K BeadChip (San Diego, CA, USA), which measures DNA methylation levels of more than 850,000 probes at single nucleotide resolution, according to the manufacturer’s recommendations. The assay involved strict quality control which described by Qiao et al*.* [23]. All the 96 samples passed quality control. The Illumina 850K Array data were processed by the Illumina BeadStudio software with default settings. Association of probes with case–control status was assessed by beta-regression models with a logistic link and associated Wald tests using R software [24]. Multiple tests were adjusted using a Bonferroni correction, with the significance threshold set at an adjusted *p* < 0.05.

### MALDI-TOF mass spectrometry

Agena matrix-assisted laser desorption ionization time-off light (MALDI-TOF) mass spectrometry (Agena Bioscience, California, USA) described by Yang et al. was utilized to quantitatively measure the methylation levels of candidate gene in two independent validations [25]. Bisulfite-converted genomic DNA was amplified by bisulfite-specific primers. The sequence of target region of *FOLR3* was showed in Additional file 1: Fig. S1. Neither the single nucleotide polymorphism (SNP) nor CpG site was in the primers. Forward primer: 5′-aggaagagagTTGAGGAAGCAGAAGTTTGAGGTTG-3′, reverse primer: 5′-cagtaatacgactcactatagggagaaggctTTATATACTCTCTCCCTCCCAAACC-3′. Upper case letters presented the sequence-specific primer regions, and non-specific tags were shown in lower case letters. DNA methylation levels were calculated on mass spectrometry by the semi-quantitative measurements at the single CpG resolution with comparing the intensities of methylated and non-methylated fragments. The methylation data were automatically collected by SpectroACQUIRE v3.3.1.3 software and visualized by EpiTyper v1.3 software.

### *FOLR3* methylation detection in NSCLC patients, NCs, and BPN cases in the validation data

To analyze the NSCLC-associated *FOLR3* methylation in peripheral blood, a 338 bp amplicon covering the *FOLR3*_CpG_1 (CpG_1, cg10533990) and *FOLR3*_CpG_2 (CpG_2, cg25634666) sites and one measurable flanking CpG site *FOLR3*_CpG_4 (CpG_4, the site couldn’t be found in the 850K assay) was designed. The methylation levels of the three measurable CpG sites were quantitatively determined in validations I and II.

### Further investigation of *FOLR3* methylation and expression in LUAD and LUSC tissues

To investigate the methylations and expressions of *FOLR3* in LUAD and LUSC tissues. The UALCAN (https://ualcan.path.uab.edu/index.html) was explored and the promoter methylations and expressions of *FOLR3* were compared between the tumor tissues and normal controls. The LUAD and LUSC datasets from TCGA were used for methylation and mRNA expression comparisons. For the protein expression comparisons, LUSC and LUAD datasets from CPTAC (https://proteomics.cancer.gov/programs/cptac) database were used.

### Further exploration of protein-drug and protein-chemical interactions of FOLR3 protein

The protein-drug and protein-chemical interactions of FOLR3 protein were investigated through NetworkAnalyst (https://www.networkanalyst.ca/). The protein and drug target information were collected from the DrugBank database and the protein-chemical information were obtained from the Comparative Toxicogenomics Database (CTD).

### Statistical analyses

All the statistical data were analyzed using R4.2.0 software. According to the histology of the tumors, NSCLC patients were divided into LUSC group and LUAD group. Kruskal–Wallis test and Mann–Whitney U test was adopted to compare the methylation levels among and between different groups/subgroups. Bonferroni correction was used and the adjusted *p* < 0.05 was considered significant. Logistic regression analysis was confirmed to be effective in identification risk factors and useful for risk model construction and risk estimation [26–28]. Here, univariable and multivariable binary logistic regression analyses were performed to analyze the associations of age, gender, *FOLR3* methylations with NSCLC patients of different histology, with the NCs and BPN cases as controls. Odd ratio (OR) and 95% confidence interval (CI) was also used to evaluate the risk of CpG_1 and CpG_2 methylations with LUSC and LUAD, with the top tertile (T3) as reference group.

To visualize and evaluate the relative LUSC and LUAD risk of the cases, nomograms were drawn with the logistic models of the variables. Receive operating curve (ROC) test was used to estimate the diagnostic power of the *FOLR3* methylations and the predicative values deduced from the multivariable logistics models. Spearman correlation analysis was used to investigate the correlations between different variables. For *FOLR3* expression comparisons, transcript per million (TPM) was used for mRNA level and z-score was used for protein level. In UALCAN, Welch’s T-test was used for comparisons between different groups or subgroups [29]. For all the analyses, *p* < 0.05 was considered statistically significant.

## Results

### Discovery of NSCLC-associated* FOLR3* hypomethylation in peripheral blood by Illumina 850K assay

An epigenome-wide screening of blood-based DNA methylation was performed in the discovering round with 48 stage I NSCLC cases and 48 cancer-free controls using Illumina 850K assay, which described by Qiao et al*.* [23]. Both CpG_1 and CpG_2 in *FOLR3* showed significantly lower methylation levels in NSCLC cases than in controls (*p*-value was 9.7 × 10^–8^ and 7.9 × 10^–8^ respectively, Additional file 1: Fig. S2). We therefore selected *FOLR3* as a candidate gene for further validation.

### Dysregulated *FOLR3* methylation levels in NSCLC patients in validation I

**Fig. 1:**
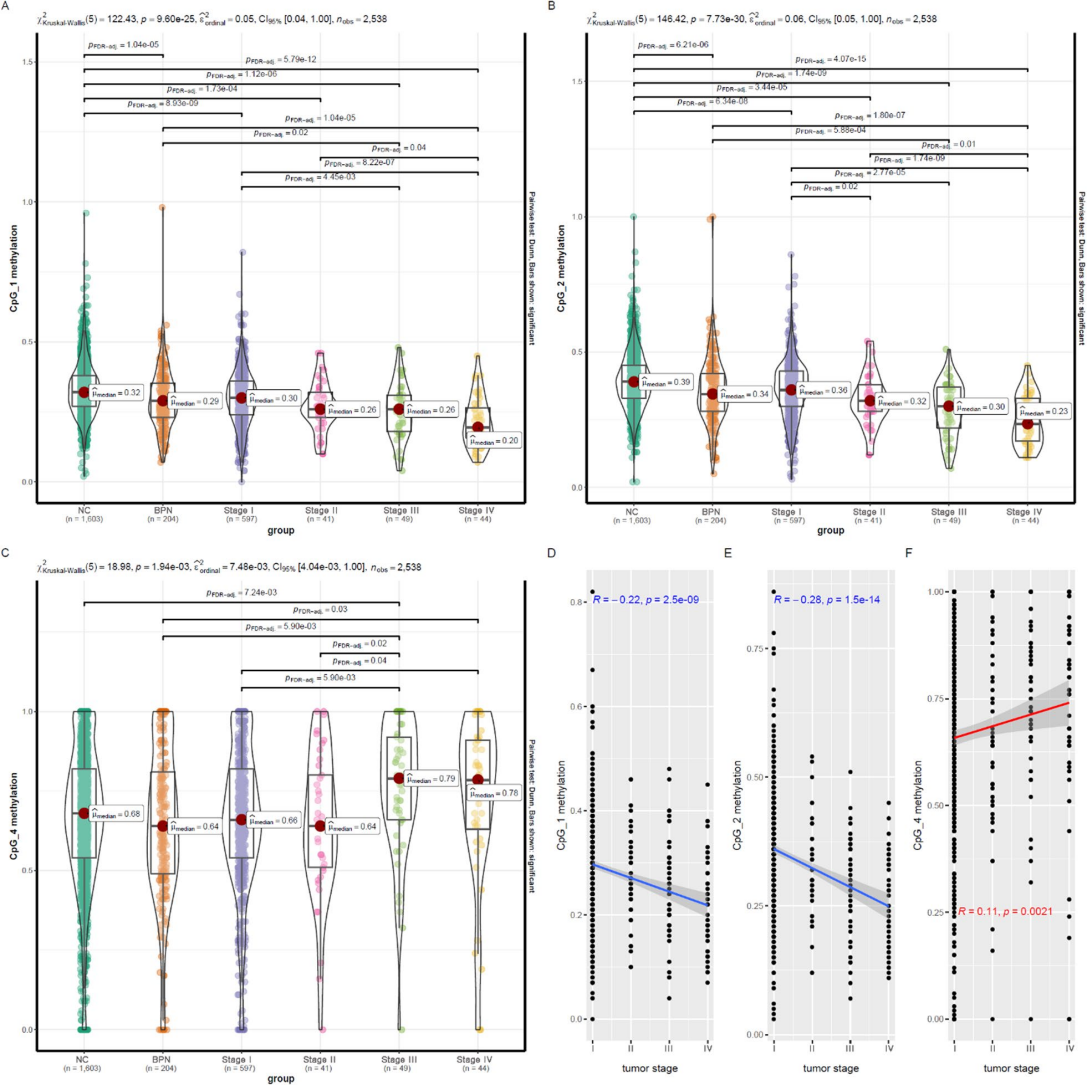
*FOLR3* methylation dysregulations in NSCLC and its associations with NSCLC stage. **A** CpG_1 methylation comparison between NSCLC patients of different stages, NCs and BPN cases. **B** CpG_2 methylation comparison between NSCLC patients of different stages, NCs and BPN cases. **C** CpG_4 methylation comparison between NSCLC patients of different stages, NCs and BPN cases. **D**, **E** Significant negative correlations of CpG_1 and CpG_2 methylations with NSCLC stage. **F** Significant positive correlations of CpG_4 methylation with NSCLC stage. Kruskal–Wallis test was used for comparisons among different groups and FDR correction was used to adjust the *p* values. Spearman correlation analysis was used to evaluate the associations between *FOLR3* methylation levels and NSCLC stage. For all the analysis, *p* < 0.05 was considered statistically significant

As shown in Fig. 1, CpG_1 (Fig. 1A) and CpG_2 (Fig. 1B) presented significant lower methylation levels in NSCLC of all the four stages than NCs. Noticeably, comparing with BPN cases, lower CpG_1 (*p* < 0.01, Fig. 1A) and CpG_2 (*p* < 0.01, Fig. 1B) methylation levels were also obvious in late-stage (stage III/IV) NSCLC patients. For CpG_4 (Fig. 1C), among the NSCLC patients, only the ones with stage III NSCLC tumors showed higher methylation level than the NCs (*p* < 0.01,). In contrast to the lower CpG_1 and CpG_2 methylations, CpG_4 presented significant higher methylation in the late-stage NSCLC patients than the BPN cases (*p* < 0.05, Fig. 1C). In consistent to their significant differences between NSCLC tumors of different stages (Fig. 1A–C), significant correlations of the methylations of CpG_1 (*R* = − 0.22, *p* < 0.01, Fig. 1D), CpG_2 (*R* = − 0.28, *p* < 0.01, Fig. 1E), and CpG_4 (*R* = 0.11, *p* < 0.01, Fig. 1F) with NSCLC stage were shown, indicating their associations with NSCLC progression.

### The heterogeneity of *FOLR3* methylation in NSCLC of different histological subtypes in validation I

**Fig. 2:**
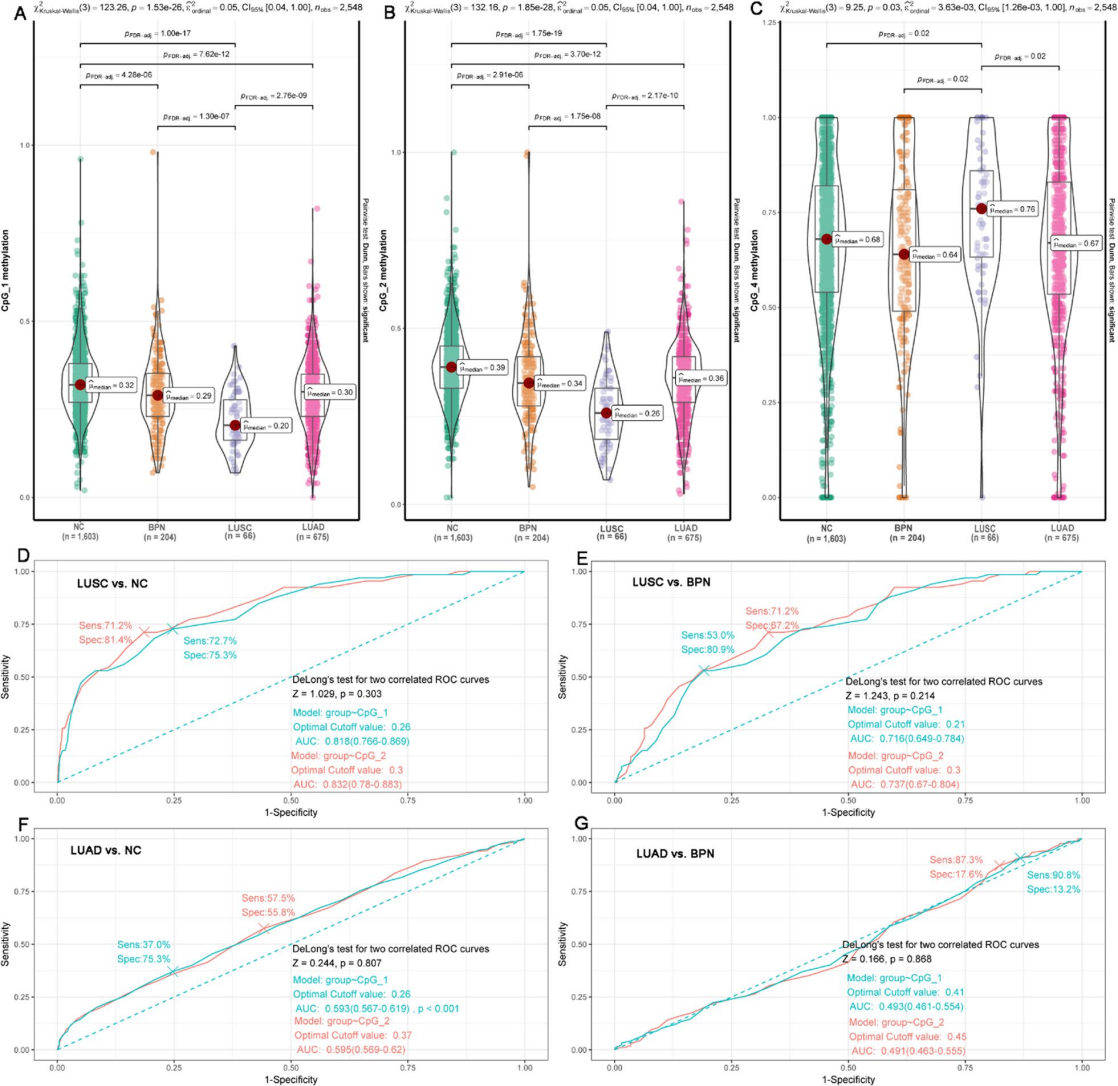
The heterogeneity and diagnostic power of *FOLR3* methylations in NSCLC of different subtypes. **A**–**C** The dysregulations and heterogeneities of *FOLR3* methylations in LUSC and LUAD. **D** The diagnostic power of CpG_1 and CpG_2 in discriminating LUSC from NCs. **E** The diagnostic power of CpG_1 and CpG_2 in discriminating LUSC from BPN. **F** The diagnostic power of CpG_1 and CpG_2 in discriminating LUAD from NCs. **G** The diagnostic power of CpG_1 and CpG_2 in discriminating LUAD from BPN. NC, NCs; PBN, benign pulmonary nodules. Kruskal–Wallis test was used for comparisons among different groups and FDR correction was used to adjust the *p* values. “multipleROC” r package was used for ROC analysis and Delong test was used for AUC comparisons. For all the analyses, *p* < 0.05 was considered significant

Although *FOLR3* methylations presented dysregulations in both LUSC and LUAD, there were significant differences between the two subtypes. As shown in Fig. 2A and B, comparing with NCs, both CpG_1 and CpG_2 presented hypomethylations in both LUSC and LUAD samples. However, for CpG_4, its hypermethylation were presented in LUSC but not in LUAD. Interestingly, lower methylation levels of both CpG_1 (Fig. 2A) and CpG_2 (Fig. 2B) while higher CpG_4 methylation (Fig. 2C) were shown in LUSC than LUAD samples. In addition, lower methylations of CpG_1 (Fig. 2A) and CpG_2 (Fig. 2B) while higher methylations of CpG_4 were shown in LUSC than BPN. However, no significant difference of *FOLR3* methylations were found between LUAD and BPN. These results indicated the heterogeneity of *FOLR3* methylation profiles in different NSCLC subtypes. Considering the associations of *FOLR3* methylations with tumor stage and their differences between LUSC and LUAD, the *FOLR3* methylation profiles in LUSC and LUAD were further investigated individually. As shown in Additional file 1: Fig. S3, comparing with NCs, the methylations of CpG_1 and CpG_2 were lower in LUSC and LUAD of all the early and late stages. While for CpG_4, its higher methylation was only shown in late-stage LUSC/LUAD. These results indicated the methylations of CpG_1 and CpG_2 might be more suitable markers for early diagnosis of LUSC and LUAD than CpG_4 methylation.

Through ROC analyses, CpG_1 and CpG_2 methylations were investigated for their diagnostic potential for LUSC and LUAD. As shown in Fig. 2D, CpG_1 and CpG_2 methylations presented to be valuable in discriminating LUSC from NCs, with AUCs of 0.818 (95%CI 0.766–0.869) and 0.832 (95%CI 0.780–0.883), respectively. With the optimal cutoff values, they could differentiate LUSC from NCs with sensitivities of 71.2% (specificity: 81.4%) and 72.7% (specificity: 75.3%), respectively. They could also discriminate LUSC from BPN well. As shown in Fig. 2E, the methylations of CpG_1 and CpG_2 could differentiate LUSC from BPN cases with AUCs of 0.716 (95%CI 0.649–0.784) and 0.737 (95%CI 0.670–0.804), respectively. Consistent with the hypomethylation of CpG_1 and CpG_2 in LUAD, their efficiency in discriminating LUAD from NCs were also indicated (Fig. 2F), with AUCs of 0.593 (95%CI 0.567–0.619) and 0.595 (95%CI 0.569–0.620), respectively. Noticeably, the DeLong tests indicated that the diagnostic power of the two CpG sites were comparable (*p* > 0.05). Noticeably, through DeLong's tests, the CpG_1 (AUC_0.818_ vs. AUC_0.593_, *p* < 0.001) and CpG_2 (AUC_0.832_ vs. AUC_0.595_, *p* < 0.001) methylations were indicated more powerful in discriminating LUSC (than LUAD) from NCs. In addition, the two CpG sites presented no significant difference between LUAD and BPN. It was not surprising to see their poor efficiency in discriminating the two groups (specificity < 20%, Fig. 2G).

### Validation of the heterogeneity and diagnostic power of *FOLR3* methylations in NSCLC of different histological subtypes in validation II

**Fig. 3:**
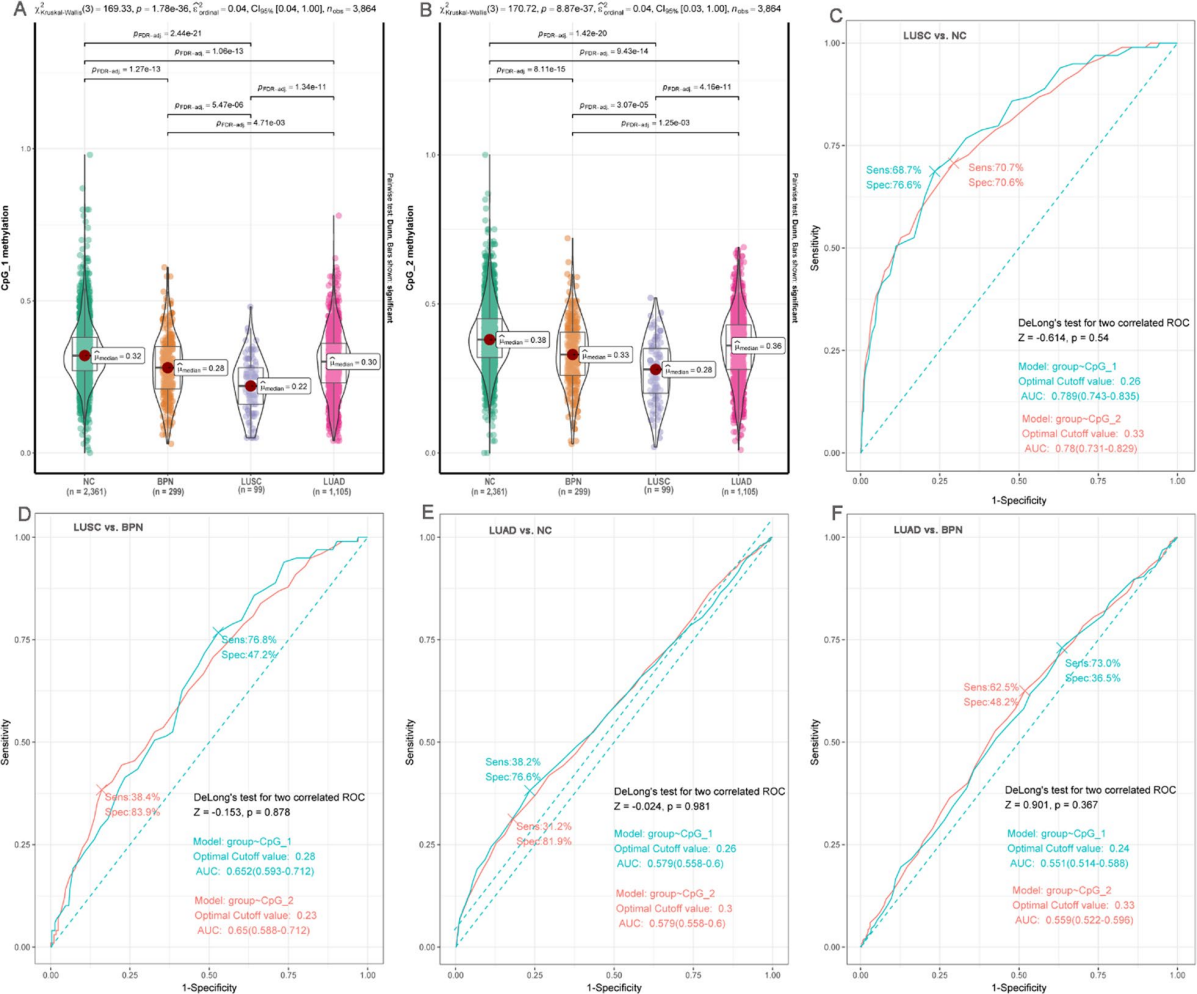
The heterogeneity and diagnostic power of *FOLR3* methylations in NSCLC dataset from Zhengzhou center. **A** The dysregulations of CpG_1 in NSCLC and its differences between different groups. **B** The dysregulations of CpG_2 in NSCLC and its differences between different groups. **C** The diagnostic power of CpG_1 and CpG_2 methylations in discriminating LUSC patients from NCs. **D** The diagnostic power of CpG_1 and CpG_2 methylations in discriminating LUSC patients from BPN cases. **E** The diagnostic power of CpG_1 and CpG_2 methylations in discriminating LUAD patients from NCs. **F** The diagnostic power of CpG_1 and CpG_2 methylations in discriminating LUAD patients from BPN cases. Kruskal–Wallis test was used for comparisons among different groups and FDR correction was used to adjust the p values. “multipleROC” r package was used for ROC analysis and Delong test was used for AUC comparisons. For all the analyses, *p* < 0.05 was considered significant

As shown in Fig. 3A, B, the differences between LUSC and LUAD were also indicated. Consistent with the results in validation I, CpG_1 and CpG_2 presented to be hypomethylated in LUSC and LUAD and their methylation levels in LUSC were obviously lower than those in LUAD. As shown in Fig. 3C, D, CpG_1 and CpG_2 methylations could discriminate LUSC from NCs and BPN cases well, with similar AUCs to the results in validation I. Similarly, the methylation levels of the two CpG sites could also differentiate LUAD from NCs (Fig. 3E), consistent with the results in validation I (Fig. 2F). Although there were slight differences of CpG_1 and CpG_2 methylations between LUAD and BPN cases in validation II (Fig. 3A, B and F), their discriminative potential presented no significant difference between validation I and validation II (*p* > 0.05, Additional file 1: Fig. S4).

### The association between hypomethylation of *FOLR3* and NSCLC stratified by variant clinical characteristics

**Fig. 4:**
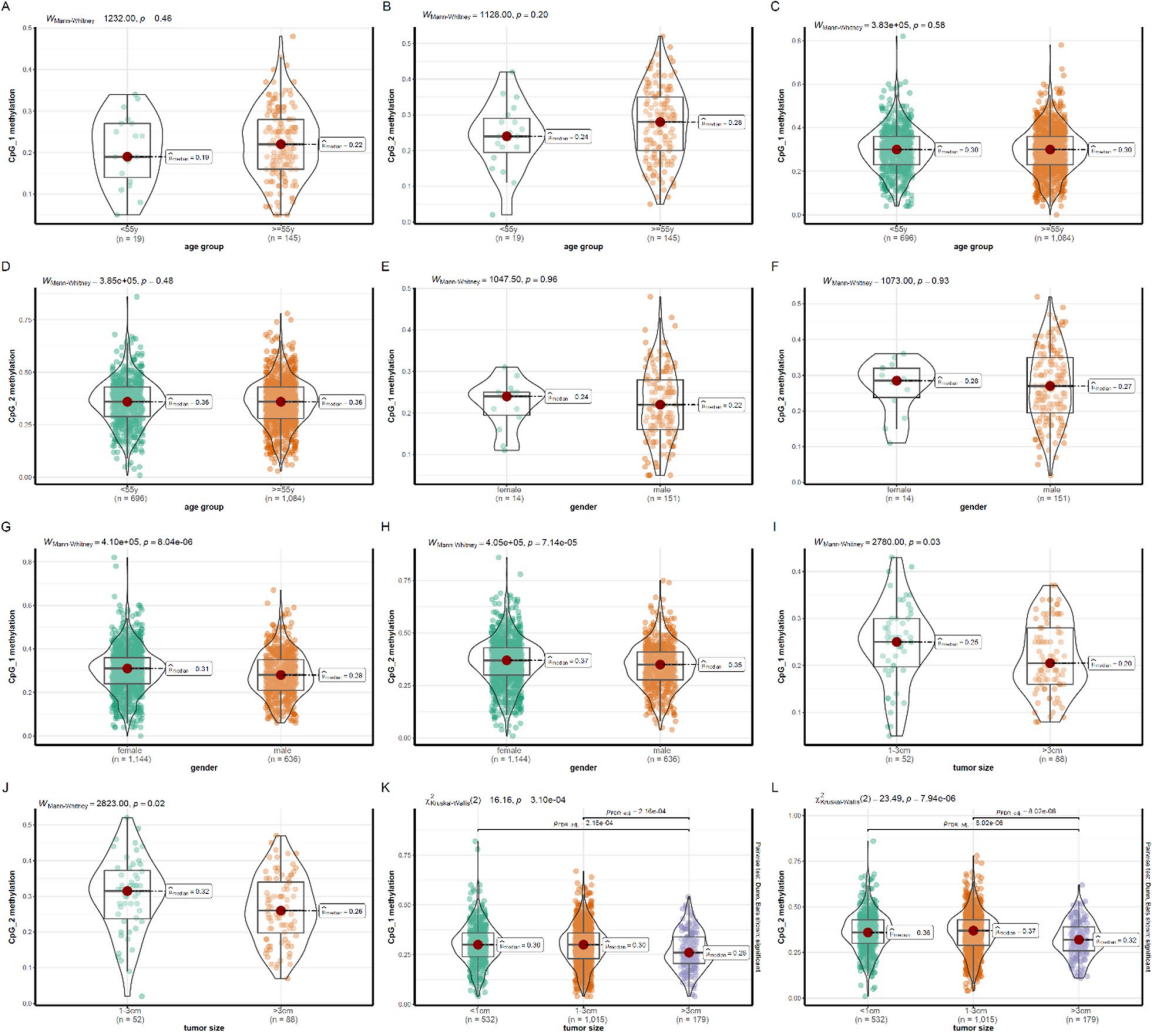
The differences of *FOLR3* methylations between NSCLC patients with different age, gender, and tumor size. **A**, **B** There was no significant difference of CpG_1 and CpG_2 methylations between LUSC patients of different age groups. **C**, **D** There was no significant difference of CpG_1 and CpG_2 methylations between LUAD patients of different age groups. **E**, **F** There was no significant difference of CpG_1 and CpG_2 methylations between female and male LUSC patients. **G**, **H** Significant lower methylations levels of CpG_1 and CpG_2 in male LUAD patients than the female ones. **I**, **J** Significant lower methylations levels of CpG_1 and CpG_2 in LUSC patients with tumor diameter > 3 cm than those with smaller tumors. **K**, **L** Significant lower methylations levels of CpG_1 and CpG_2 in LUAD patients with tumor diameter > 3 cm than those with smaller tumors. Mann–Whitney test and Kruskal–Wallis were used for comparisons and *p* < 0.05 was considered statistically significant

As the results from validation I and validation II were consistent, we combined the two datasets to explore the associations of the methylation levels of CpG_1 and CpG_2 with different clinical features. As gender and age were shown to play important roles in the patterns of DNA methylation [30, 31], here, we also investigated their potential roles in *FOLR3* methylations and the methylation levels of CpG_1 and CpG_2 were compared between different gender and age groups. As shown in Fig. 4A–D, no significant difference of CpG_1 and CpG_2 methylations were shown between different age groups of LUSC and LUAD patients. However, in contrast to the similar methylation levels of CpG_1 (Fig. 4E) and CpG_2 (Fig. 4F) methylations between female and male LUSC patients, lower methylation levels of CpG_1 (Fig. 4G) and CpG_2 (Fig. 4H) were shown in male LUAD patients than the female ones. These results indicated the different effects of gender on *FOLR3* methylations in LUSC and LUAD. With regard to the relationship between tumor size and *FOLR3* methylations, the tumors with diameter > 3 cm presented lower methylation levels of CpG_1 and CpG_2 than the smaller tumors both in LUSC (Fig. 4I, J) and LUAD (Fig. 4K, L). Consistent with their correlations with tumor stage in the two validations, these results also indicated with tumor progression.

### Risk model for LUSC and LUAD in combined validation data

**Fig. 5:**
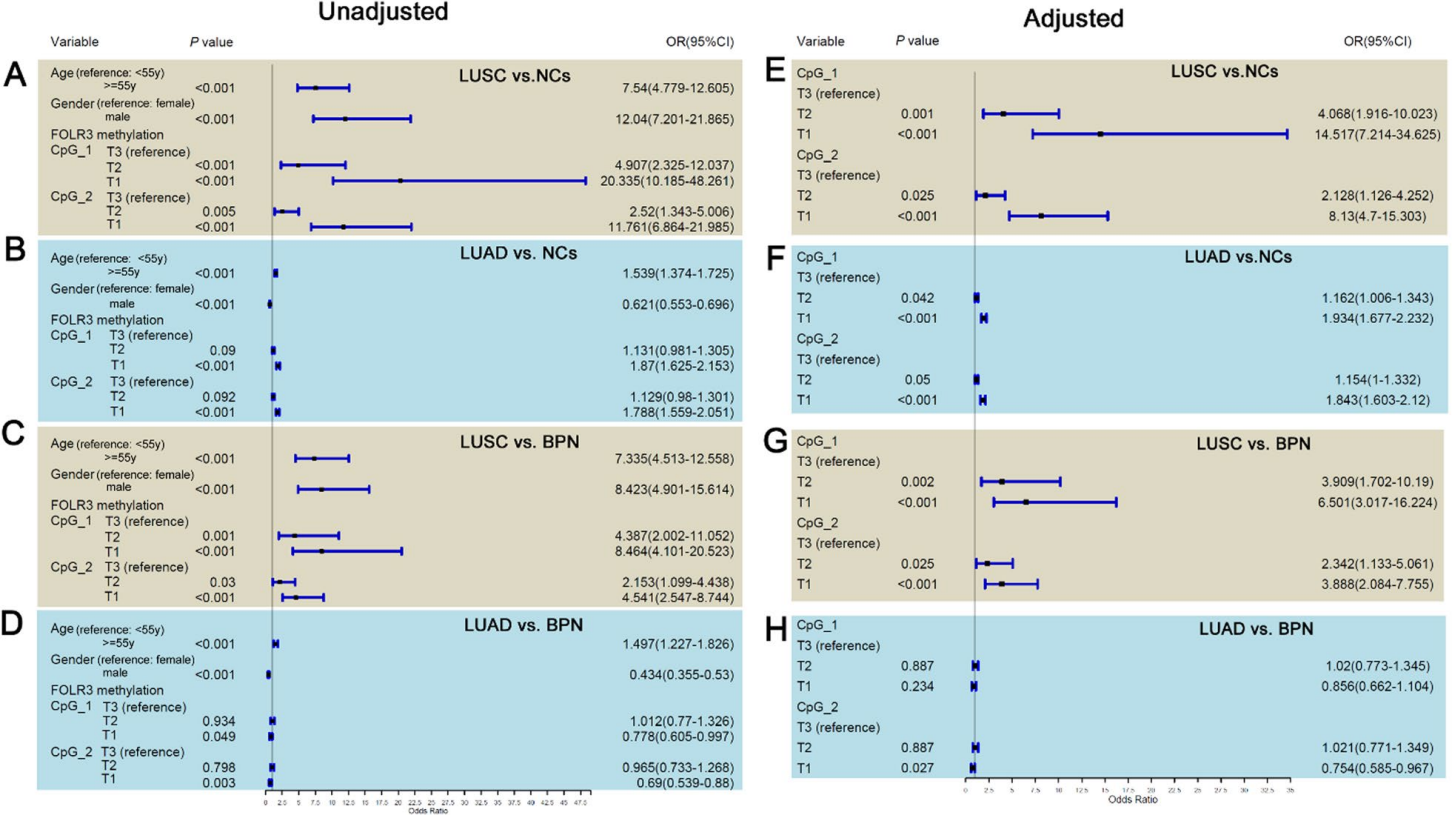
The associations of *FOLR3* methylations with LUSC and LUAD risk. **A**, **B** The association of age, gender and *FOLR3* methylations with LUSC and LUAD risk in NCs. **C**, **D** The association of age, gender and *FOLR3* methylations with LUSC and LUAD risk in BPN cases. **E**, **F** The age and gender corrected associations of *FOLR3* methylations with LUSC and LUAD risk in NCs. **G**, **H** The age and gender corrected associations of *FOLR3* methylations with LUSC and LUAD risk in BPN cases. For CpG_1, T1, T2 and T3 represented the methylation levels of ≤ 0.27, higher than 0.27 while no more than 0.35, and > 0.35, respectively. For CpG_2, T1, T2, and T3 indicated the methylation levels of ≤ 0.33, higher than 0.33 while no more than 0.41, and > 0.41 respectively. Univariable (**A**–**D**) and multivariable (**E**–**H**) logistic regression analyses were used and *p* < 0.05 was considered significant

Through univariable logistic regression analyses, the associations of age, gender, CpG_1 and CpG_2 methylations with LUSC and LUAD were evaluated. Although no significant correlation between age and *FOLR3* methylation was shown in Fig. 4A–D**,** the associations of age with LUSC and LUAD were indicated. As shown in Fig. 5A, B, age ≥ 55y was shown to be a risk indicator for LUSC and LUAD, with ORs of 7.54 (95%CI 4.779–12.605) and 1.539 (95%CI 1.374–1.725), respectively. In contrast to the consistent effects of aging on LUSC and LUAD risk, male was shown to be a risk indicator for LUSC (OR: 12.04, 95%CI 7.201–21.885) while a protective factor for LUAD (OR: 0.621, 95%CI 0.553–0.696), indicating the opposite effects of gender on LUSC and LUAD occurrence (Fig. 5A, B). For *FOLR3* methylations, with the top tertiles as the reference groups, the middle tertile (T2) and the bottom tertile (T1) of CpG_1 and CpG_2 methylations were all presented to be associated with a higher risk for LUSC, suggesting that CpG_1 and CpG_2 hypomethylations were risk indicators for LUSC (Fig. 5A). While for their associations with LUAD risk, only the T1 of the CpG_1 and CpG_2 methylations were indicated to be risk factors (Fig. 5B).For BPN cases, their age, gender and CpG_1 and CpG_2 methylations levels were also investigated for their associations with LUSC and LUAD risk (Fig. 5C, D). Consistent with the results in NCs, aging also seemed to be a risk factor for LUSC and LUAD occurrence. With age < 55y as reference, age ≥ 55y could increase the LUSC risk and LUAD risk of the BPN cases with 6.335 folds (Fig. 5C) and 49.7% (Fig. 5D). Interestingly, the opposite associations of gender with LUSC and LUAD risk were also shown in BPN cases. Male was indicated to be a risk factor for LUSC (Fig. 5C, OR > 1, *p* < 0.001) while a protective factor for LUAD (Fig. 5D, OR < 1, *p* < 0.001). Noticeably, for BPN cases, CpG_1 and CpG_2 hypomethylations levels presented positive associations with LUSC risk (OR > 1, *p* < 0.001) while negative relations to LUAD risk (OR < 1, *p* < 0.05). When the effects were adjusted with age and gender, the effects of CpG_1 and CpG_2 also existed (Fig. 5E–H), indicating their independents relations with NSCLC. Obviously, the associations of CpG_1 and CpG_2 methylations with LUSC (Fig. 5E and G) were also larger than their relations with LUAD (Fig. 5F and H).

**Fig. 6:**
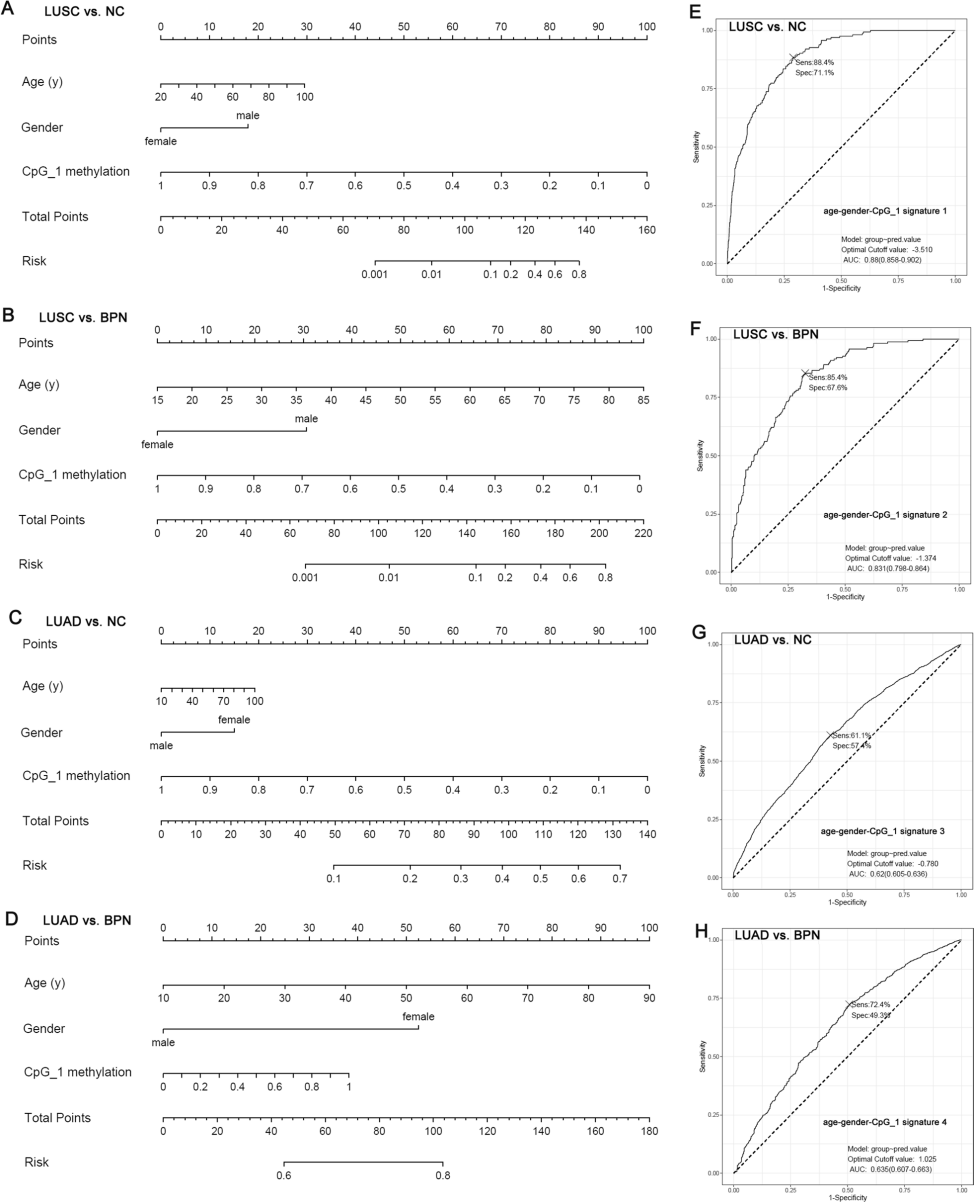
The nomograms and ROCs of the risk models (signatures). **A**–**D** The nomograms of the age-gender-CpG_1 signatures. **E**, **F** The ROCs of the age-gender-CpG_1 signature 1–4 for discriminating LUSC and LUAD from NCs and BPN cases. Nomogram analysis was performed with “rms” package in R. Logistic regression analysis and ROC analysis were used and *p* < 0.05 was considered statistically significant

Multivariable logistic regression analysis was performed to construct risk models for discrimination of LUSC and LUAD patients from NCs and BPN cases, with the significant variables in Fig. 5. As there was a strong correlation between CpG_1 and CpG_2 (Additional file 1: Fig. S5), only CpG_1 was used for further analysis to avoid multicollinearity. With age, gender and CpG_1 as arguments, the risk models (age-gender- CpG_1 signature 1–4) were constructed and the nomograms were shown (Fig. 6A–D). The coefficients of the variables in the four signatures were shown in Additional file 1: Table S2. It was shown that the age-gender-CpG_1 signature 1 could discriminate LUSC from NCs with AUC of 0.880 (95%CI 0.858–0.902). At an optimal cutoff value of -3.510, the sensitivity and the specificity were 88.4% and 71.1%, respectively (Fig. 6E). Similarly, the age-gender-CpG_1 signature 2 could also discriminate LUSC from BPN cases well, with AUC of 0.831 (0.798–0.864) (Fig. 6F). As shown in Fig. 6G, H, the age-gender-CpG_1 signature 3 and age-gender-CpG_1 signature 4 could discriminated LUAD from NCs and BPN cases with AUCs of 0.620 (95%CI 0.605–0.632) and 0.635 (95%CI 0.607–0.663), respectively. In addition, the nomograms in Fig. 6A and C also showed that the CpG_1 methylation status had the greatest weighting and stronger power for discriminating LUSC and LUAD from NCs. While, when discriminating LUSC and LUAD from BPN cases, age was indicated to have similar weighting with CpG_1 methylation (Fig. 6F) or greatest weighting among all the variables (Fig. 6G).

### *FOLR3* methylations and expressions in LUAD and LUSC tissues

As shown in Additional file 1: Fig. S6A, no significant difference of *FOLR3* promoter methylation was shown between LUAD tissues and their normal controls, inconsistent with the *FOLR3* hypomethylation in LUAD blood. Different races and age groups presented no significant differences of *FOLR3* promoter methylation in LUAD tissues (Additional file 1: Figs. S6B and D), indicating that race and age have no significant impacts on *FOLR3* promoter methylation in LUAD tissues. In contrast, the male patients were shown to have lower FOLR3 promoter methylations in LUAD tissues than female patients (Additional file 1: Fig. S6C). Similarly, significant difference of FOLR3 promoter methylation was shown between LUAD patients with different smoking status (Additional file 1: Fig. S6E) and TP53 mutation status (Additional file 1: Fig. S6F). It was indicated that smoking history and TP53 mutation were associated with *FOLR3* promoter methylation in LUAD tissues.

For LUSC, as shown in Additional file 1: Fig. S7A, *FOLR3* promoter presented a significant lower methylation in the tumor tissues than the normal controls, consistent with the hypomethylation of *FOLR3* in LUSC blood. For the LUSC patients of different races, African-American were shown to have significant lower *FOLR3* promoter methylation than Caucasian patients in their tumor tissues (Additional file 1: Fig. S7B). In contrast, no significant difference of FOLR3 promoter methylation were shown in LUSC tumors between different gender (Additional file 1: Fig. S7C) and age groups (Additional file 1: Fig. S7D). Similar to LUAD tissues, LUSC tissues with smoking history were shown to be shown to lower *FOLR3* promoter methylation than the non-smoker patients (Additional file 1: Fig. S7E). However, no significant difference of *FOLR3* promoter methylation between LUSC tissues with and without TP53 mutation (Additional file 1: Fig. S7F).

For the *FOLR3* expressions, both LUAD tissues and LUSC tissues were shown to have lower *FOLR3* expression than their normal controls, both at mRNA level (Additional file 1: Fig. S8A, B) and protein level (Additional file 1: Fig. S8C, D).

### Protein-chemical and protein drug interactions of FOLR3

**Fig. 7:**
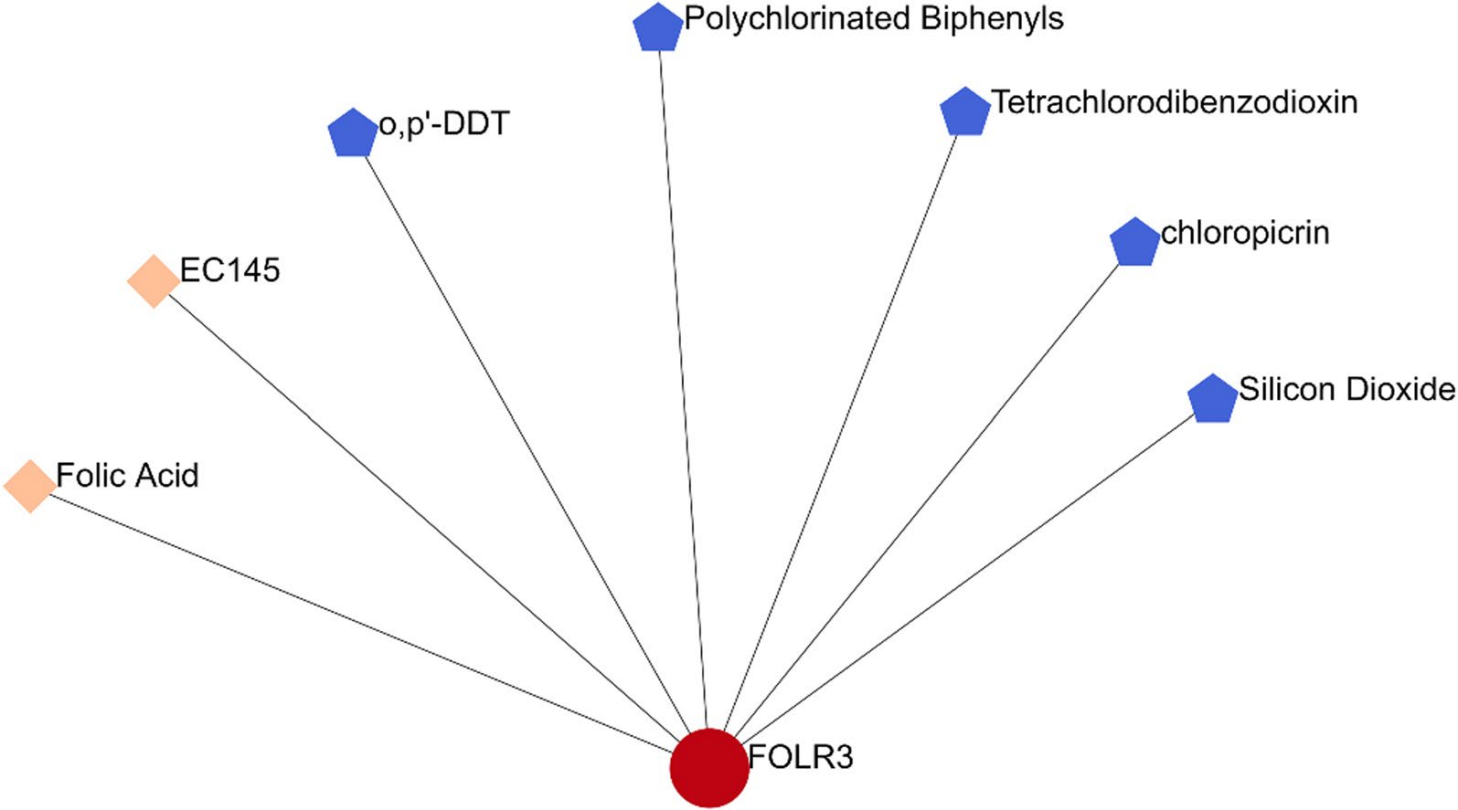
The protein-chemical and protein drug interactions of FOLR3

As shown in Fig. 7, FOLR3 protein was shown to be a target of two drugs (folic acid and EC145) in the Drugbank database. And five chemicals (o,p′-DDT, Polychlorinated Biphenyls, tetrachlorodibenzodioxin, chloropicrin, and Silicon Dioxide) presented to have interactions with FOLR3 proteins. These drugs and chemicals should be considered in LUAD and LUSC treatment.

## Discussion

In this study, we found NSCLC-associated *FOLR3* hypomethylation in peripheral blood by epigenome-wide screening using Illumina 850K assay. The strong association between blood-based *FOLR3* hypomethylation at two CpG sites (CpG_1 and CpG_2) with NSCLC were further confirmed via mass spectrometry in two independent case–control studies with over 6000 subjects from different clinical centers. The CpG_1 and CpG_2 methylations could discriminate LUSC and LUAD patients from NCs well in two validations. LUSC presented lower CpG_1 and CpG_2 methylations than LUAD, indicating the heterogeneity of *FOLR3* methylations between different NSCLC histological types. In contrast to LUAD, LUSC also could be discriminated from BPN cases well by CpG_1 and CpG_2 methylations, highlighting the stronger associations of *FOLR3* methylation with LUSC. The larger AUCs of CpG_1 and CpG_2 in discriminating LUSC from NCs and BPNs also indicated that* FOLR3* hypomethylation may be more effective for the detection of LUSC.

The folic acid receptors (FOLRs) have three isotypes (FOLR1, FOLR2 and FOLR3) in human. FOLR1 and FOLR2 are located in the cell membrane surface and transported folic acid into cell membrane using glycosylphosphatidylinositol (GPI)-anchored membrane protein by endocytosis. FOLR3 is mainly expressed in haematopoietic tissues, such as spleen and bone marrow, and its protein products are primarily secreted [32, 33]. Previous studies have shown significant overexpression of FOLR1 in various tumor types of epithelial origin, including lung, pancreatic, colorectal, gastric, kidney, bladder, breast, ovarian, endometrial, testicular, brain and neck cancers, compared with cognate normal tissues [34–41]. Its prognostic roles in breast, colorectal, ovarian and endometrial cancers [40, 42, 43] were reported and its tumor specificity makes it an attractive target of prognosis and therapy. However, little is known about the clinical value of FOLR3 in human cancers.

In the present study, all NSCLC samples were collected before biopsy, surgery and any cancer related treatment, approximately 80% of the NSCLC cases were at stage I. Our data, therefore, showed a significant association between *FOLR3* hypomethylation in peripheral blood and increased the LUSC and LUAD risk of the cases, even at a very early stage (stage I), indicating the altered DNA methylation signatures as a potential biomarker for the early detection of NSCLC. In fact, both in the two validations, the CpG_1 and CpG_2 methylations presented their diagnostic potential in discriminating LUSC and LUAD patients from NCs. When adjusted with age and gender, CpG_1 and CpG_2 also presented significant associations with LUSC and LUAD, indicating its independent relations to NSCLC.

Considering the similar results in the two validations, the two datasets were combined. In the whole dataset, gender was shown to be associated with CpG_1 and CpG_2 methylations in LUAD, but not in LUSC, indicated the heterogeneity of the associations between gender and DNA methylation profiles between different histological subtypes of NSCLC. The gender-related DNA methylation differences in cancers have been reported previously. Qiao et al. reported that the *SH3BP5* hypermethylation was associated with male gender in the peripheral blood of LC patients [44]. It is well documented that inherent DNA methylation differences in peripheral blood between male and female exist in many CpG sites, which can be partly attributed to the difference in circulating sex hormone [45]. Methylation pattern can also be affected by lifestyles and environment factors [46, 47]. Therefore, the differences of sex hormone as well as the behavior differences between genders may explain the different patterns of gender-associated *FOLR3* methylation in LUSC and LUAD.

Moreover, there was significant negative correlations between NSCLC stage and the methylation levels of CpG_1 and CpG_2. *FOLR3* hypomethylation was more significant in larger tumors, both in LUSC and LUAD. As *FOLR3* hypomethylation was presented to be more significant in larger LUAD/LUSC tumors (> 3 cm) than the smaller ones, a significant association of *FOLR3* hypomethylation with tumor proliferation and progression could be deduced. Since larger tumors often indicate worse prognosis, the association of *FOLR3* hypomethylation with the prognosis of LUAD and LUSC patients can be deduced.

To improve the prognosis of the NSCLC patients, early detection is crucial. Combination of several variables were confirmed to be able to improve the diagnostic efficiency in many studies [48, 49]. Here, age, gender and *FOLR3* methylation were shown to be independent indicators for LUSC and LUAD risk. Considering the strong positive correlations between CpG_1 methylation and CpG_2 methylations and their comparable efficiency in differentiating LUSC and LUAD from NCs and BPN case, we choose CpG_1 methylation to represent the methylation level of *FOLR3*. With age, gender, and CpG_1 methylation, we constructed four risk signatures to evaluate the risk scores of the samples. It was shown that the age-gender-CpG_1 signature could discriminate LUSC from NCs and BPN with AUCs of 0.888 and 0.831, higher than the diagnostic potential of CpG_1 methylation and CpG_2 methylation individually. These results suggested the good performance of age-gender-CpG_1 signature in LUSC diagnosis. As age and gender were general information and blood could be obtained easily, the age-gender-CpG_1 signature might be new practical indicator for LUSC diagnosis. As for LUAD, although the discriminating power of CpG_1 and CpG_2 methylations and the signatures were not so high, they could combine with other indicators and improve the diagnostic power for LUAD.

We also investigated the *FOLR3* promoter methylation in LUAD and LUSC tissues. In contrast to the hypomethylation of *FOLR3* in LUAD blood, no significant difference of *FOLR3* promoter methylation between LUAD tumors and normal tissues were shown. Although consistence of *FOLR3* hypomethylation in LUSC tissues and LUSC blood were shown, the beta value seemed to be lower in the blood samples. These results indicating that circulating tumors cells might not be the main source of *FOLR3* hypomethylation in LUAD and LUSC blood. We also investigated the potential impacts of race, gender, age and smoking status on *FOLR3* methylation in LUAD and LUSC tissues. In both LUAD tissues and LUSC tissues, smoking was shown to be associated with *FOLR3* methylation. As a risk factor for LC, especially for LUSC, we speculated that smoking might be associated with *FOLR3* hypomethylation in LUAD and LUSC blood.

There were also limitations for our study. Firstly, we focused on the dysregulation of *FOLR3* methylation and its diagnostic potential in this study. As for whether *FOLR3* hypomethylation is the cause or result of NSCLC is unclear, we will conduct an in-depth analysis and exploration of it in future research. Secondly, although the association of *FOLR3* hypomethylation with NSCLC progression could be deduced, the prognostic values of *FOLR3* methylation couldn’t be estimated due to the insufficiency of follow-up data. Thirdly, as the samples in this study were all from China, there might be limitations for the results to be applied to other regions and further study is needed to validate the findings in a broader population. Finally, the roles of *FOLR3* methylation in *FOLR3* expression regulation needed to be explored in further studies.

## Conclusions

In summary, we revealed and validated the strong association between the blood-based hypomethylation of *FOLR3* and the very early-stage NSCLC patients in a large-scale case–control study from different clinical centers. The strong associations of *FOLR3* hypomethylation with LUSC were highlighted. *FOLR3* methylation and its combination with age and gender might be new useful markers for LUSC diagnosis and new candidates for combination to improve LUAD diagnostic efficiency.

### Supplementary Information

**Additional file 1: Figure S1.** Sequence of the FOLR3 amplicon. The FOLR3 amplicon examined by mass spectrometry (chr11:71846595-71846932, sense strand, build 37/hg19, in the UCSC Genome Browser). The three measurable CpG sites are highlighted, and the one undetectable CpG site is underlined.** Figure S2.** FOLR3 hypomethylations in NSCLC in the discovery dataset. **Figure S3.** The differences of FOLR3 methylations between normal controls and NSCLC patients of different stages. **Figure S4.** Discriminative efficiency comparisons of FOLR3 methylations in differentiating LUAD from BPNs in the validations. **Figure S5. **Correlations between CpG_1 methylation and CpG_2 methylation in NSCLC. **Figure S6.** *FOLR3* promoter methylation in LUAD tissues. **A** *FOLR3* promoter methylation comparison between LUAD tissues and normal controls. **B**–**F** *FOLR3* promoter methylation comparison between LUAD tissues of different races, gender, age groups, smoking status, and TP53 mutation status, respectively. **Figure S7.** *FOLR3* promoter methylation in LUSC tissues. **A** *FOLR3* promoter methylation comparison between LUSC tissues and normal controls. **B**–**F** *FOLR3* promoter methylation comparison between LUSC tissues of different races, gender, age groups, smoking status, and TP53 mutation status, respectively. **Figure S8**. *FOLR3* expressions in LUAD and LUSC at mRNA level and protein level. **A**, **B** FOLR3 was down-regulated in LUAD and LUSC at mRNA level. **C**, **D** FOLR3 was down-regulated in LUAD and LUSC at protein level. **Table S1.** The clinical features of the samples in validation I and II. **Table S2.** Multi-variable logistic regression analysis of age, gender and *FOLR3* methylation in NSCLC.

## Data Availability

The datasets used and/or analyzed during the current study are available from the corresponding author on reasonable request.

## Abbreviations

LC: Lung cancer
FOLR3: Folate receptor gamma gene
SCLC: Small cell lung cancer
NSCLC: Non-small cell lung cancer
LUAD: Lung adenocarcinoma
LUSC: Lung squamous cell carcinoma
NCs: Normal controls
BPN: Benign pulmonary nodule
LDCT: Low-dose computed tomographic
MALDI-TOF: Matrix-assisted laser desorption ionization time-of-flight
OR: Odd ratio
CI: Confidence interval
ROC: Receiver operating character
AUC: Area under the curve
