# Supplementary material for: Blood FOLR3 methylation dysregulations and heterogeneity in non-small lung cancer highlight its strong associations with lung squamous carcinoma
Source: Respir Res. 2024 Jan 25;25:59. doi: 10.1186/s12931-024-02691-8 (PMC10809478; doi:10.1186/s12931-024-02691-8)
Supplement: Supplementary file 1 — Additional file 1: Figure S1. Sequence of the FOLR3 amplicon. The FOLR3 amplicon examined by mass spectrometry (chr11:71846595-71846932, sense strand, build 37/hg19, in the UCSC Genome Browser). The three measurable CpG sites are highlighted, and the one undetectable CpG site is underlined. Figure S2. FOLR3 hypomethylations in NSCLC in the discovery dataset. Figure S3. The differences of FOLR3 methylations between normal controls and NSCLC patients of different stages. Figure S4. Discriminative efficiency comparisons of FOLR3 methylations in differentiating LUAD from BPNs in the validations. Figure S5. Correlations between CpG_1 methylation and CpG_2 methylation in NSCLC. Figure S6. FOLR3 promoter methylation in LUAD tissues. A FOLR3 promoter methylation comparison between LUAD tissues and normal controls. B–F FOLR3 promoter methylation comparison between LUAD tissues of different races, gender, age groups, smoking status, and TP53 mutation status, respectively. Figure S7. FOLR3 promoter methylation in LUSC tissues. A FOLR3 promoter methylation comparison between LUSC tissues and normal controls. B–F FOLR3 promoter methylation comparison between LUSC tissues of different races, gender, age groups, smoking status, and TP53 mutation status, respectively. Figure S8. FOLR3 expressions in LUAD and LUSC at mRNA level and protein level. A, B FOLR3 was down-regulated in LUAD and LUSC at mRNA level. C, D FOLR3 was down-regulated in LUAD and LUSC at protein level. Table S1. The clinical features of the samples in validation I and II. Table S2. Multi-variable logistic regression analysis of age, gender and FOLR3 methylation in NSCLC. [file 12931_2024_2691_MOESM1_ESM.docx]

**Additional file Figures**

CTGAGGAAGCAGAAGCCTGAGGCTGTCTAGAGTCTCACTCCTGCATCAGCAGGCCACCACCTGTGGTTCCTCCTTGTGCAAATTTGAAAAGAATTGCATAAAACACTGGAGAAATCCAAGAGGGGAAGTCCACAAGGGCGGTGGCTCCCTACAAGGTCACAGAGCAAGCTGGTGTCAGAGCCTGGACCTACAGCGCTGTTGGTGGAGGTCCTGCCTCCAGGTAGGGGAAGGGCTCCCTCTCACCTCTACACGCAGCGCATTTCTTGGCTCAGCTGCCCTGTAGGGGATGCAGGGTGGGGACAGCAGAGATCTGGGCCTGGGAGGGAGAGAGTACACAA

**Figure S1.** Sequence of the *FOLR3* amplicon. The *FOLR3* amplicon examined by mass spectrometry (chr11:71846595-71846932, sense strand, build 37/hg19, in the UCSC Genome Browser). The three measurable CpG sites are highlighted, and the one undetectable CpG site is underlined.


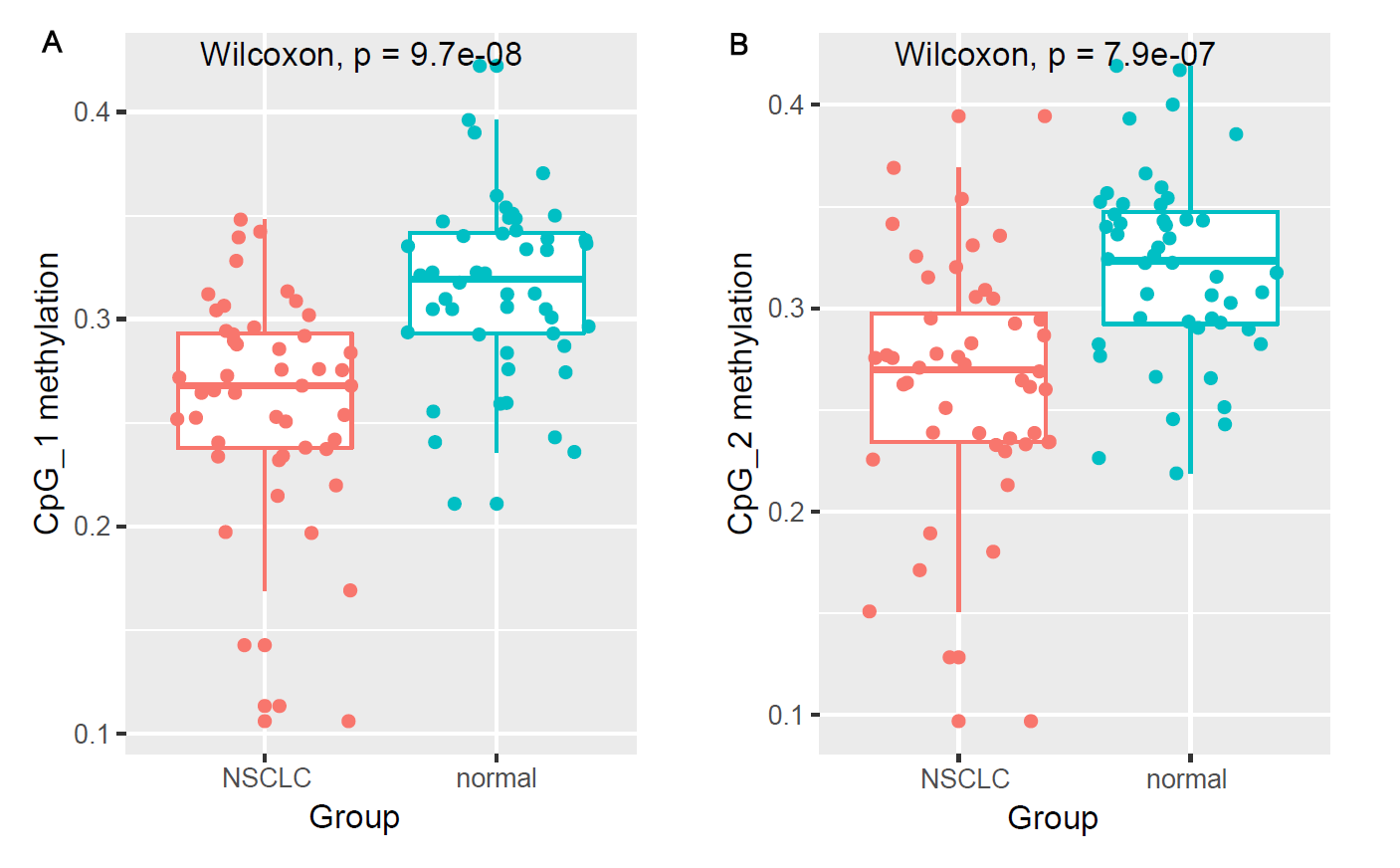


**Figure S2.** *FOLR3* hypomethylations in NSCLC in the discovery dataset. (A) Lower CpG_1 methylation in NSCLC than normal controls. (B) Lower CpG_2 methylation in NSCLC than normal controls. Wilcoxon test was used for comparisons and *p* < 0.05 was considered significant.


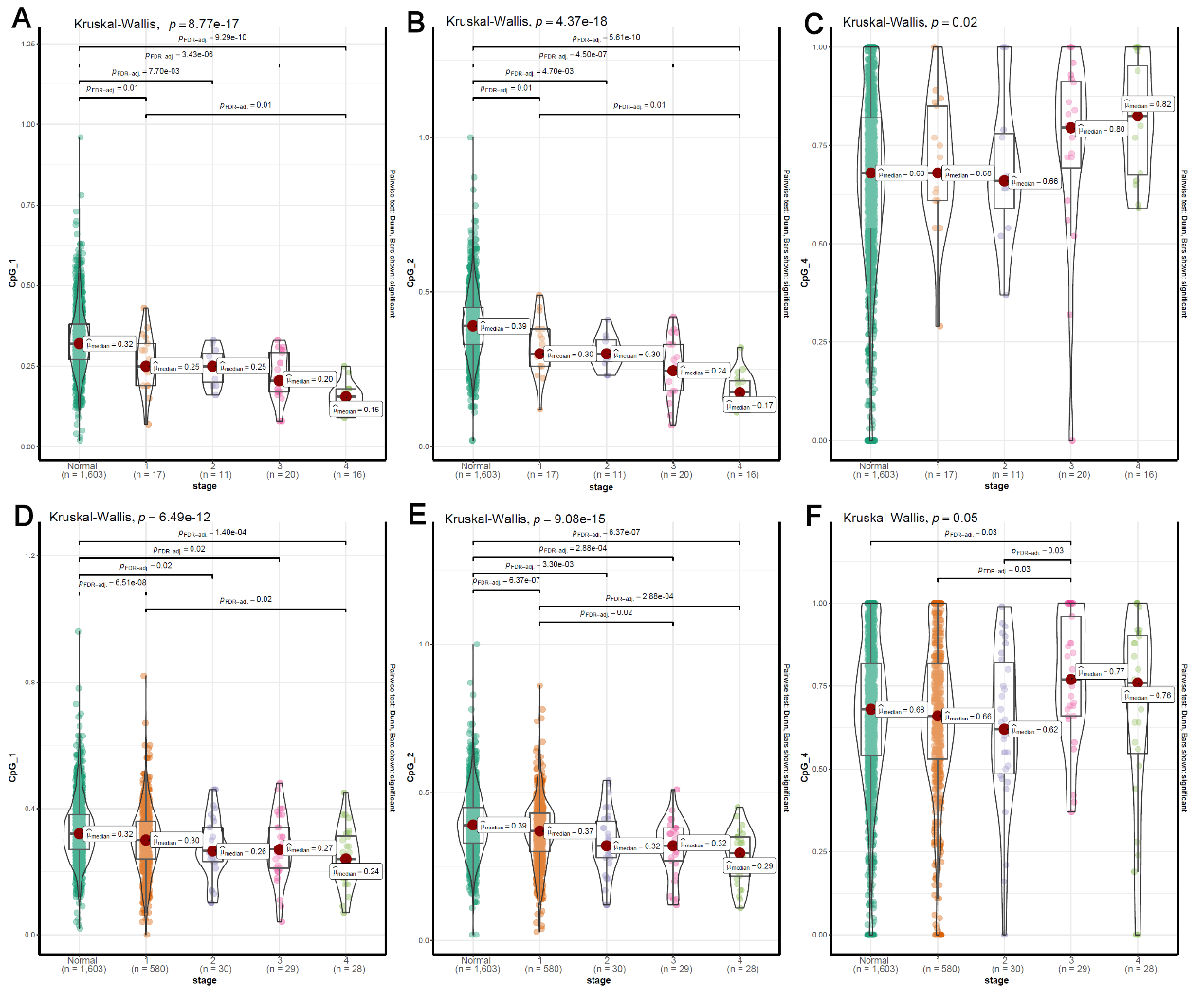


**Figure S3.** The differences of *FOLR3* methylations between normal controls and NSCLC patients of different stages. (A-B) Comparing with NCs, the methylations of CpG_1 and CpG_2 were lower in LUSC of all the early and late stages. (C) No significant difference of CpG_4 methylation between NCs and LUSC of different stages. (D-E) Comparing with NCs, the methylations of CpG_1 and CpG_2 were lower in LUAD of all the early and late stages. (F) Higher CpG_4 methylation late-stage III LUAD. Stage 1-4 represented stage I-IV, respectively. Kruskal-Wallis test was used for comparisons among different groups and FDR correction was used to adjust the *p* values. *P* < 0.05 was considered significant.


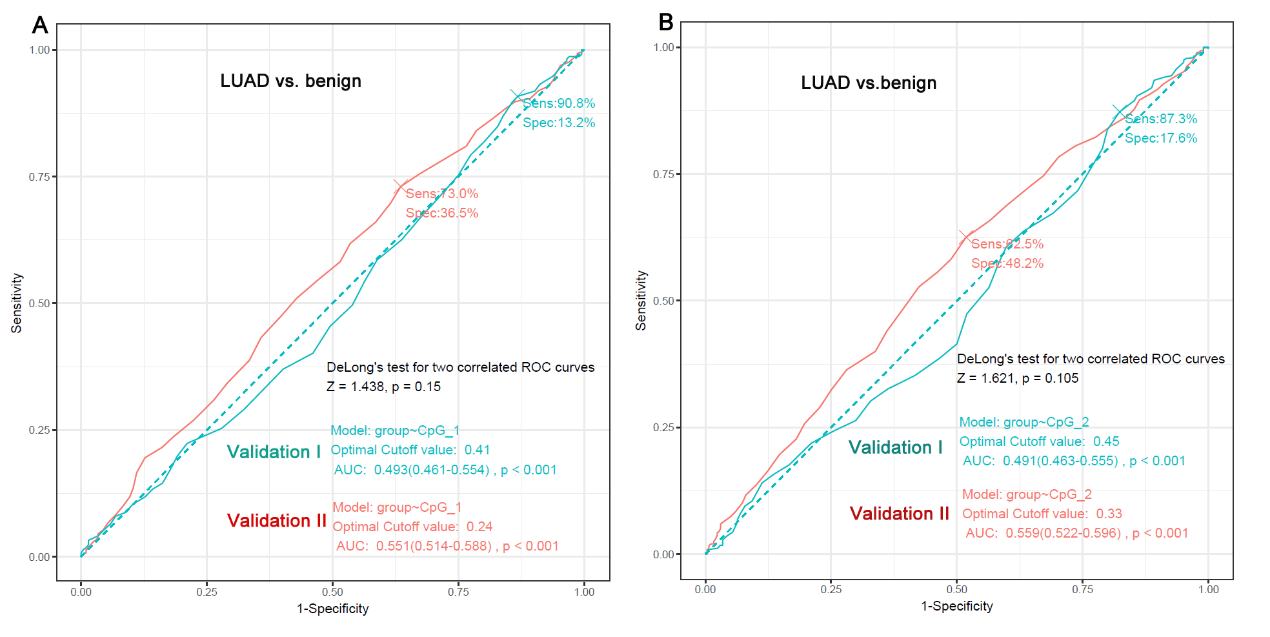


**Figure S4.** Discriminative efficiency comparisons of *FOLR3* methylations in differentiating LUAD from BPNs in the validations. (A) CpG_1 methylation presented no significant differences of its efficiency in discriminating LUAD patients from BPN cases between validations I and II. (B) (A) CpG_2 methylation presented no significant differences of its efficiency in discriminating LUAD patients from BPN cases between validations I and II.


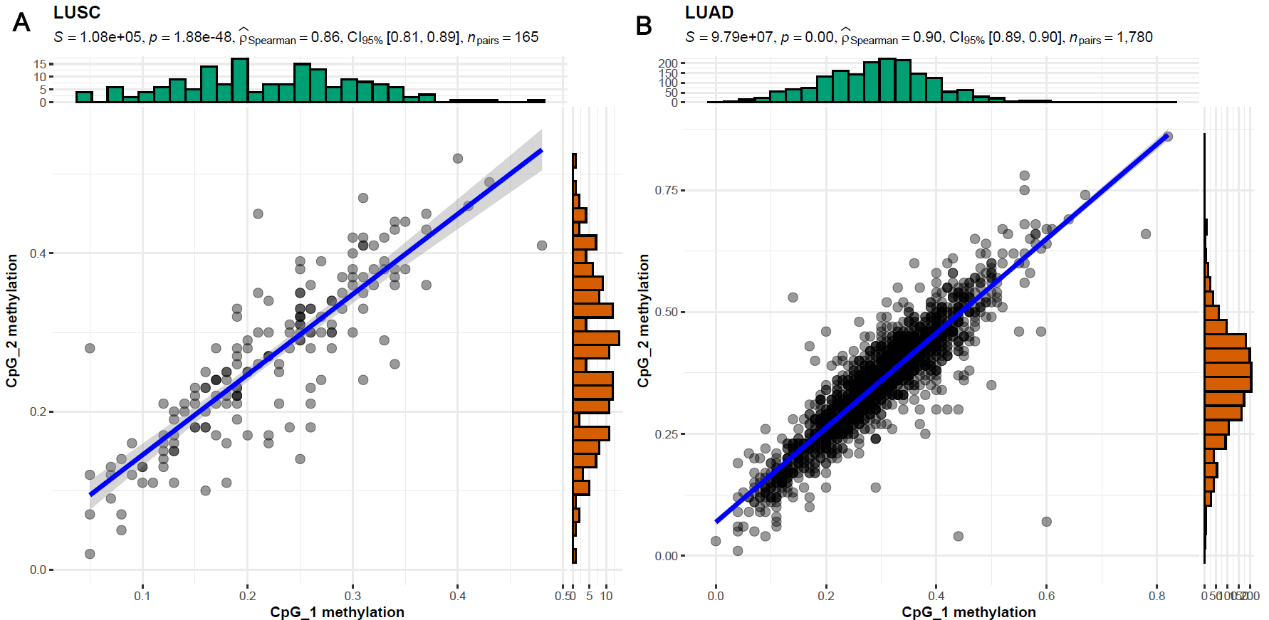


**Figure S5.** The positive correlations between CpG_1 methylation and CpG_2 methylation in NSCLC. (A) The positive correlations between CpG_1 methylation and CpG_2 methylation in LUSC. (B) The positive correlations between CpG_1 methylation and CpG_2 methylation in LUAD. Spearman correlation analysis was used and p<0.05 was considered significant.


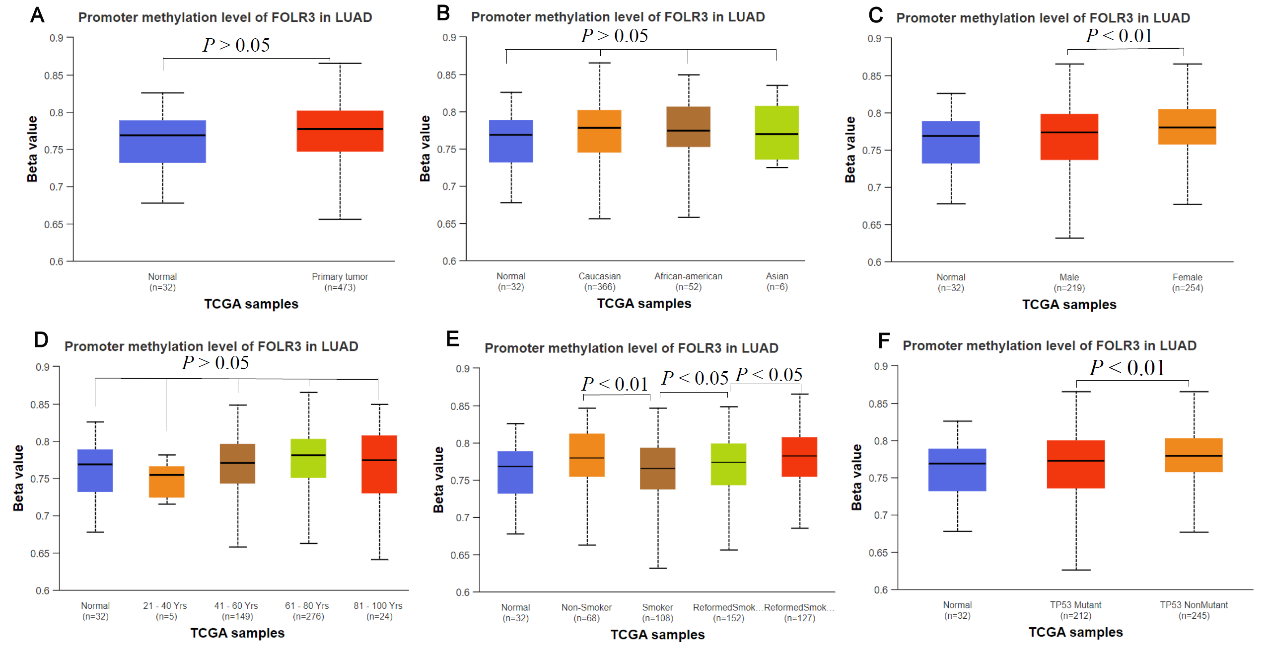


**Figure S6.** *FOLR3* promoter methylation in LUAD tissues. (A) *FOLR3* promoter methylation comparison between LUAD tissues and normal controls. (B-F) *FOLR3* promoter methylation comparison between LUAD tissues of different races, gender, age groups, smoking status, and TP53 mutation status, respectively.


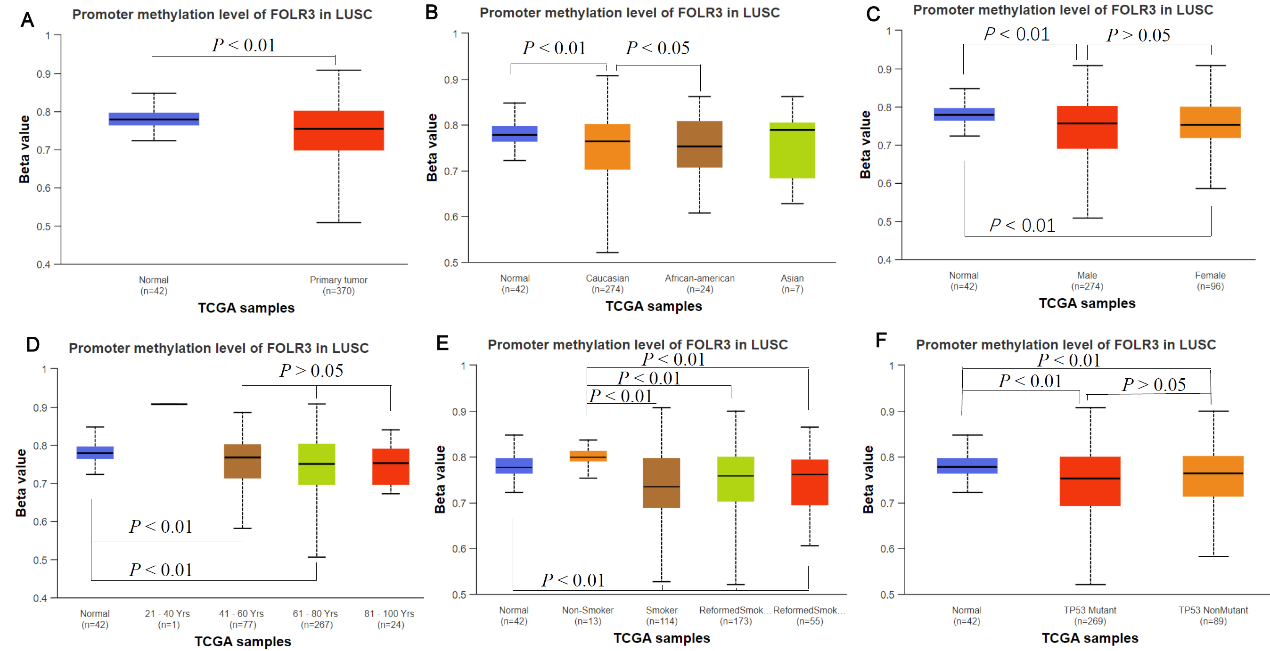


**Figure S7.** *FOLR3* promoter methylation in LUSC tissues. (A) *FOLR3* promoter methylation comparison between LUSC tissues and normal controls. (B-F) *FOLR3* promoter methylation comparison between LUSC tissues of different races, gender, age groups, smoking status, and TP53 mutation status, respectively.


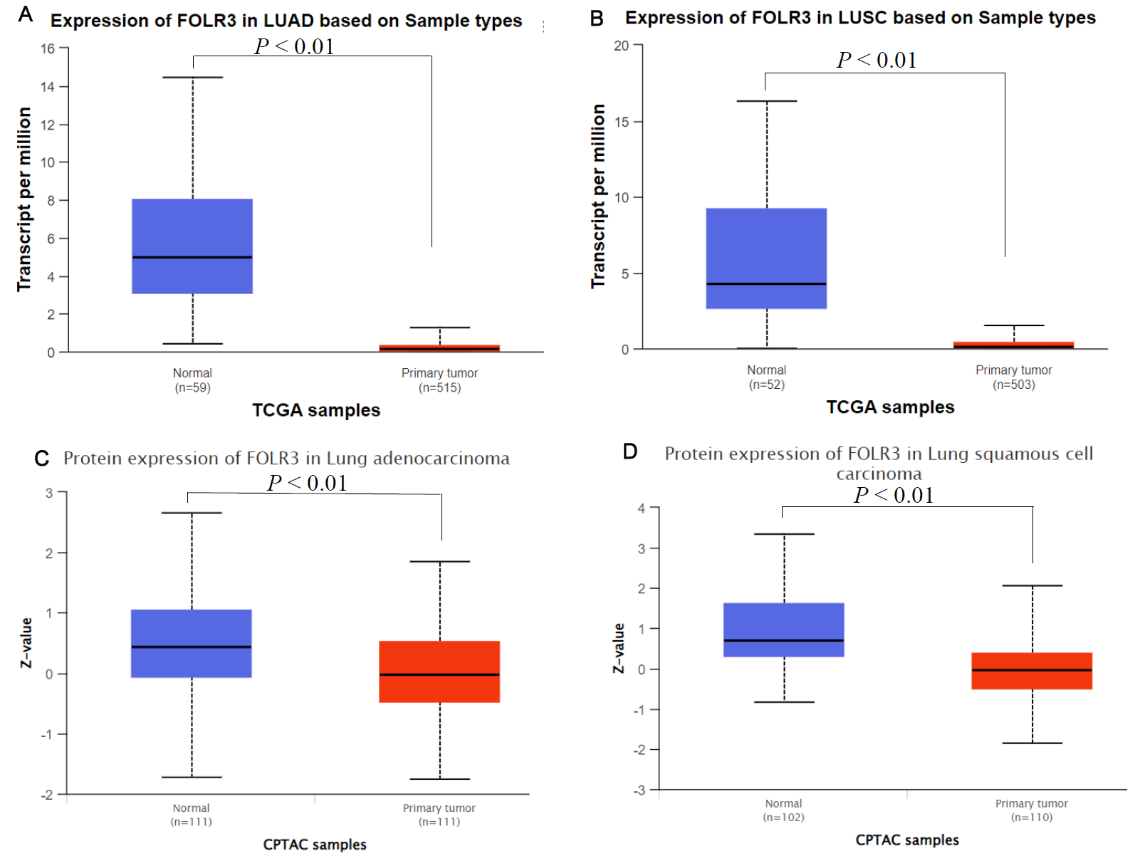


**Figure S8**. *FOLR3* expressions in LUAD and LUSC at mRNA level and protein level. (A-B) FOLR3 was down-regulated in LUAD and LUSC at mRNA level. (C-D) FOLR3 was down-regulated in LUAD and LUSC at protein level.

Table S1. The clinical features of the samples in validation I and II.

| Subgroup | Validation I (n=2548) | | |  | Validation II (n=3864) | | |
| --- | --- | --- | --- | --- | --- | --- | --- |
|  | NSCLC  N=741 | NC  N = 1603 | BPN  N = 204 |  | NSCLC  N=1204 | NC  N = 2361 | BPN  N = 299 |
| Age (y) |  |  |  |  |  |  |  |
| <55y | 271 (36.6%) | 801 (50.0%) | 103 (50.5%) |  | 444 (36.9%) | 1169(49.5%) | 144 (48.2%) |
| >=55y | 469 (63.4%) | 802 (50.0%) | 101 (49.5%) |  | 760 (63.1%) | 1192 (50.5%) | 155 (51.8%) |
| Gender |  |  |  |  |  |  |  |
| Male | 299 (40.4%) | 745 (46.5%) | 117 (57.4%) |  | 488 (40.5%) | 1127 (47.7%) | 165 (55.2%) |
| Female | 442 (59.6%) | 858 (53.5%) | 87 (42.6%) |  | 716 (59.5%) | 1234 (52.3%) | 134 (44.8%) |
| Histology |  |  |  |  |  |  |  |
| LUAD | 675 (91.1%) |  |  |  | 1105 (91.8%) |  |  |
| LUSC | 66 (8.9%) |  |  |  | 99 (8.2%) |  |  |
| Adenoma |  |  | 12(5.9%) |  |  |  | 18 (6.0%) |
| Granuloma |  |  | 25 (12.3%) |  |  |  | 40 (13.4%) |
| Tuberculosis |  |  | 14 (6.9%) |  |  |  | 19 (6.4%) |
| Inflammatory  pseudotumor |  |  | 7 (3.4%) |  |  |  | 8 (2.7%) |
| Hamartoma |  |  | 12 (5.9%) |  |  |  | 16 (5.4%) |
| Chronic inflammation |  |  | 67 (32.8%) |  |  |  | 98 (32.8%) |
| Mycotic infection |  |  | 13 (6.4%) |  |  |  | 18 (6.0%) |
| Sclerotic pulmonary cytoma |  |  | 4 (2.0%) |  |  |  | 7 (2.3%) |
| Pulmonary fibrosis |  |  | 16 (7.8%) |  |  |  | 23 (7.7%) |
| Fibroma |  |  | 2 (1.0%) |  |  |  | 4 (1.3%) |
| Leiomyoma |  |  | 2 (1.0%) |  |  |  | 3 (1.0%) |
| Others/unknown |  |  | 30 (14.7%) |  |  |  | 45（15.1%） |
| Tumor stage |  |  |  |  |  |  |  |
| Stage I | 597 (80.6%) |  |  |  | 969 (80.5%) |  |  |
| Stage II | 41 (5.5%) |  |  |  | 59 (4.9%) |  |  |
| Stage III | 49 (6.6%) |  |  |  | 94 (7.8%) |  |  |
| Stage IV | 44 (5.9%) |  |  |  | 73 (6.1%) |  |  |
| NA | 10 (1.3%) |  |  |  | 9 (0.7%) |  |  |
| Tumor size |  |  |  |  |  |  |  |
| <1cm | 206 (27.8%) |  | 122 (59.8%) |  | 326 (27.1%) |  | 178 (59.5%) |
| 1-3 cm | 402 (54.3%) |  | 52 (25.5%) |  | 665 (55.2%) |  | 80 (26.8%) |
| >3cm | 104 (14.0%) |  | 9 (4.4%) |  | 163 (13.5%) |  | 14 (4.7%) |
| NA | 29 (3.9%) |  | 21 (10.3%) |  | 50 (4.2%) |  | 63 (21.1%) |

NA, not available.

**Table S2.** Multi-variable logistic regression analysis of age, gender and *FOLR3* methylation in LUSC and LUAD.

| LUSC vs. NCs | | | | | |
| --- | --- | --- | --- | --- | --- |
|  | Estimate | Std. Error | z value | *P* value | OR (95%CI) |
| Intercept | -4.173 | 0.629 | -6.639 | *P*<0.001 | 0.015 (0.004-0.052) |
| Age | 0.046 | 0.008 | 5.931 | *P*<0.001 | 1.047 (1.031-1.063) |
| Gender (reference: female) |  |  |  |  |  |
| Male | 2.211 | 0.286 | 7.735 | *P*<0.001 | 9.127 (5.401-16.706) |
| CpG_1 methylation | -12.340 | 1.076 | -11.471 | *P*<0.001 | 4.371*10^-6^(5.119*10^-7^-3.487*^10-5^) |
| LUSC vs. BPN | | | | | |
|  | Estimate | Std. Error | z value | *P* value | OR (95%CI) |
| Intercept | -6.248 | 0.821 | -7.609 | *P*<0.001 | 0.002 (0.0004-0.009) |
| Age | 0.087 | 0.012 | 7.457 | *P*<0.001 | 1.091 (1.067-1.117) |
| Gender (reference: female) |  |  |  |  |  |
| Male | 1.873 | 0.308 | 6.079 | *P*<0.001 | 6.507 (3.668-12.376) |
| CpG_1 methylation | -6.057 | 1.198 | -5.056 | *P*<0.001 | 0.002 (0.0002-0.023) |
| LUAD vs. NCs | | | | | |
|  | Estimate | Std. Error | z value | *P* value | OR (95%CI) |
| Intercept | 0.136 | 0.174 | 0.782 | 0.434 | 1.146 (0.814-1.614) |
| Age | 0.008 | 0.002 | 3.293 | 0.001 | 1.008 (1.003-1.013) |
| Gender (reference: female) |  |  |  |  |  |
| male | -0.554 | 0.060 | -9.210 | *P*<0.001 | 0.575 (0.510-0.646) |
| CpG_1 methylation | -3.692 | 0.319 | -11.561 | *P*<0.001 | 0.025 (0.013-0.046) |
| LUAD vs. BPN | | | | | |
|  | Estimate | Std. Error | z value | *P* value | OR (95%CI) |
| Intercept | 0.329 | 0.296 | 1.113 | 0.266 | 1.389 (0.780-2.485) |
| Age | 0.021 | 0.004 | 4.703 | *P*<0.001 | 1.021 (1.012-1.030) |
| Gender (reference: female) |  |  |  |  |  |
| Male | -0.874 | 0.104 | -8.368 | *P*<0.001 | 0.417 (0.340-0.512) |
| CpG_1 methylation | 0.636 | 0.508 | 1.252 | 0.210 | 1.888 (0.701-5.129) |

Multi-variable logistic regression analysis was used and *p* < 0.05 was considered significant. LUSC, lung squamous carcinoma; LUAD, lung adenocarcinoma; NC, normal control; BPN, benign pulmonary nodule; OR, odd ratio.
